# E‐cigarette support for smoking cessation: Identifying the effectiveness of intervention components in an on‐line randomized optimization experiment

**DOI:** 10.1111/add.16294

**Published:** 2023-07-16

**Authors:** Catherine Kimber, Vassilis Sideropoulos, Sharon Cox, Daniel Frings, Felix Naughton, Jamie Brown, Hayden McRobbie, Lynne Dawkins

**Affiliations:** ^1^ London South Bank University London UK; ^2^ IOE, UCL's Faculty of Education and Society University College London London UK; ^3^ Department of Behavioural Science and Health University College London London UK; ^4^ School of Health Sciences University of East Anglia Norwich UK; ^5^ National Drug and Alcohol Research Centre University of New South Wales Sydney NSW Australia

**Keywords:** Digital interventions, e‐cigarettes, multi‐phase optimization strategy (MOST), nicotine, smoking cessation, smoking reduction, tailored advice, tobacco, vaping

## Abstract

**Aims, Design and Setting:**

The aim of this study was to determine which combination(s) of five e‐cigarette‐orientated intervention components, delivered on‐line, affect smoking cessation. An on‐line (UK) balanced five‐factor (2 × 2 × 2 × 2 × 2 = 32 intervention combinations) randomized factorial design guided by the multi‐phase optimization strategy (MOST) was used.

**Participants:**

A total of 1214 eligible participants (61% female; 97% white) were recruited via social media.

**Interventions:**

The five on‐line intervention components designed to help smokers switch to exclusive e‐cigarette use were: (1) tailored device selection advice; (2) tailored e‐liquid nicotine strength advice; (3): tailored e‐liquid flavour advice; (4) brief information on relative harms; and (5) text message (SMS) support.

**Measurements:**

The primary outcome was 4‐week self‐reported complete abstinence at 12 weeks post‐randomization. Primary analyses were intention‐to‐treat (loss to follow‐up recorded as smoking). Logistic regressions modelled the three‐ and two‐way interactions and main effects, explored in that order.

**Findings:**

In the adjusted model the only significant interaction was a two‐way interaction, advice on flavour combined with text message support, which increased the odds of abstinence (odds ratio = 1.55, 95% confidence interval = 1.13–2.14, *P* = 0.007, Bayes factor = 7.25). There were no main effects of the intervention components.

**Conclusions:**

Text‐message support with tailored advice on flavour is a promising intervention combination for smokers using an e‐cigarette in a quit attempt.

## INTRODUCTION

The annual global burden of tobacco smoking includes more than 6 million deaths [1]. Even with the most effective treatments (combined pharmacotherapy + behavioural support), quit rates remain modest [2, 3]. In England, e‐cigarettes (EC) have become the most popular methods of quitting over the last 10 years [4], supporting approximately 35% of all attempts [5]. There is increasing evidence for their efficacy in promoting quitting over more conventional cessation aids; between 8 to 12% of those making a quit attempt using a nicotine EC typically achieve abstinence at 6 months, compared with 6% of those using nicotine‐replacement therapy (NRT), or 4% of those using behavioural support only or no support [6]. In stop smoking services between 2020 and 2021, approximately 65% of quit attempts involving EC (alone or combined with medication), were successful at 4‐week follow‐up compared with 59% of attempts not involving EC [7]. However, while data from throughout Great Britain shows that, at a population level, approximately three in five smokers report having tried an EC, approximately 65% discontinue use and continue smoking [8]. A recent Cochrane Review [9] pooling randomized controlled trials (RCTs) and uncontrolled observation studies show higher quit rates among those allocated to a nicotine EC condition compared to non‐nicotine EC [relative risk (RR) = 1.94], NRT (RR = 1.53) or behavioural support (RR = 2.61) at 6 months. This would translate to an additional three to six quitters per 100. Given their potential effectiveness and the harm reduction benefits if people fully switch [10], a clearer understanding of what types of support can help more smokers to fully transition to e‐cigarettes is warranted.

RCTs often include multi‐component approaches (provision of medication/product, support/advice on use, monitoring, counselling, etc.) and disentangling effects of individual intervention components or interactions between them is challenging. Understanding the contribution of specific intervention components before packaging optimized components into an RCT should maximize therapeutic efficacy using robust empirical design. While this approach has been recently utilized in mainstream smoking cessation [11, 12, 13, 14], at the time of writing it has not been used to identify promising intervention components in studies using EC for smoking cessation.

The current study used a factorial design guided by the multi‐phase optimization strategy (MOST) [14, 15] to screen multiple intervention components and identify effective components (or combinations) that could be packaged into a novel, scalable, digital intervention to support smokers who are obtaining an EC on‐line to quit smoking. Between 13 and 19% of smokers report purchasing on‐line [16, 17], with indication of future growth [18], and EC starter kits appear to be the most frequently purchased product [18]. However, little guidance or support on what or how to use is currently provided. We identified five intervention components which may meet this need: (1) tailored advice (TA) on EC device, (2) TA on nicotine strength, (3) TA on flavour, (4) brief information on relative harms and (5) text message support [short message service (SMS)]. The rationale for choosing these interventions is summarized below.

First, the wide range of devices available can be confusing, with ‘too much choice’ [19] or difficulty finding the right combination of EC device, nicotine strength and flavour as barriers to EC initiation [20]. Evidence suggests that second‐ and third‐generation devices can outperform early ‘cigalike’ and disposable models [21, 22, 23], but such devices are sometimes described as too ‘bulky’ or ‘scary’ [24], which may act as a deterrent.

Secondly, lack of satisfaction from EC use appears to be the chief reason for discontinuing use. In a 2021 survey in Great Britain, 79% of smokers who had tried but discontinued EC use reported vaping as less satisfying than smoking. There is likely to be a variety of reasons for inadequate satisfaction, although insufficient nicotine delivery and strength may play a role. Given that EC nicotine concentration is associated with improvements in smoking cessation [9], advice to choose a nicotine strength that adequately satisfies tobacco craving, based on nicotine dependency, may help to support sustained use.

Thirdly, flavour is a commonly cited reason for EC use and is known to enhance their appeal [25, 26], as well as promoting cessation [27], although whether specific flavours or advice on which flavour to select increase the effectiveness of EC for smoking cessation is unknown. Although many vape shops offer support and assistance around product choice and e‐liquid nicotine strength and flavour, for some this can feel overwhelming [28], and this support is usually absent when purchasing on‐line. Smokers purchasing on‐line may therefore benefit from simple tailored advice on which device, nicotine strength and flavour to choose in order to help them transition more easily and fully to EC.

Fourthlt, a key barrier to initiating or sustaining EC use is concern over safety. A growing number of people in England and elsewhere in the world consider EC to be equally or more harmful than smoking [19] and safety concerns are frequently cited among smokers as reasons for not trying or discontinuing EC [19]. Providing information around the relative harms of EC compared with smoking may help to correct such misperceptions and promote use to support smoking cessation. In support of this, a recent experimental study has shown that a nicotine fact sheet can help to correct EC risk perceptions [29] and messages conveying reduced risk information around EC have been associated with lower odds of smoking [30].

Finally, difficulties with starting using an EC have been reported as challenging for some smokers [19, 24, 31]; therefore, more support in the early days of transitioning may be needed. To address this, we have developed a set of text messages [32] designed to assist a successful transition to vaping by providing ongoing technical assistance and behavioural support. Mobile phone text messages have been shown to increase long‐term abstinence among smokers by approximately 70% [33, 34] and is recommended as a cost‐effective option by the World Health Organization (WHO) [35].

### Aims and objectives

The aim of the study was to test promising intervention components among smokers interested in using an EC to support an attempt to quit smoking.

### Primary objective

The primary objective was to determine which of five intervention component(s): (1) TA on EC device, (2) TA on nicotine strength, (3) TA on flavour, (4) brief information on relative harms and (5) text message support, or their interactions, affect 4‐week abstinence at 12 weeks post‐randomization.

### Secondary objectives

To examine the differences between intervention components on:

1. 7‐day point‐prevalence abstinence at 12 weeks post‐randomization
2. Proportion of people reporting a 50% reduction or more in baseline cigarette consumption at 12 weeks post‐randomization
3. To test adherence and compliance with the intervention components

These objectives were pre‐registered in the study protocol [36].

## METHODS

### Design

The study utilized an efficient 2 × 5 factorial design that enabled the assessment of five interventions, described below, with the same sample size required to examine a single intervention with identical statistical power [37, 38]. Permutations of ON and OFF of the five intervention components resulted in 32 experimental conditions (see Supporting information, Table S1). Data were collected over a period of 6 months, with approximately 46 participants randomized to one of the 32 permutations at baseline. All participants were followed‐up at 12 weeks, with 33 to 44 participants assigned to each of the permutations (see Supporting information, Table S1).

### Participants

The study was advertised through Facebook and Reddit, with a call for smokers interested in using an EC to try to quit and an incentive of a voucher (for the value of £50) towards the purchase of an EC kit. Participants were included if they were aged 18 years or over, daily smoker, UK resident, fluent in English, interested in quitting, interested in using an EC, currently have access to a mobile phone and able to make an on‐line purchase. Participants were excluded if they were daily EC users, unable or unwilling to be contacted in 3 months’ time or were affiliated with the tobacco or e‐cigarette industries.

#### Sample size calculation

Based on Holliday *et al*. [39], we estimated a 3‐month abstinence rate of 16% (without any advice or information), that tailoring on nicotine strength, flavour or device could increase abstinence rates to 20.8% and brief information could increase abstinence to 19.2% [40]. To detect two‐tailed differences between 16% (control) and 19% (brief information) with a power of 0.80 at α = 0.05, a total sample of 1184 was required. This sample size allowed the detection of differences between two halves of 2 × 2 interactions (total *n* = 592) with odds ratio (OR) = ≥ 1.34 with the same power/α parameters. Although we did not power the study on three‐factor or higher interactions, three‐way interactions were tested to define which two‐way and main effects to include in the model (see Primary analysis, below).

### Study settings/procedure

Data were collected on‐line between April and October 2020. From the study webpage, participants were directed to the Qualtrics baseline survey to check eligibility. Eligible participants were invited to provide electronic informed consent before completing the baseline survey. After completing baseline measures, participants were randomly allocated to one of the 32 conditions. Conditions were randomized in Qualtrics using the Randomizer function, each generated an equal number of times (see Supporting information, Table S1).

Personalized recommendations regarding the interventions allocated were displayed at the end of the baseline survey and sent via e‐mail together with detailed instructions (see Supporting information, S9) and a direct link to an on‐line EC store (Totally Wicked) with a unique voucher code, purchased by the study team, to receive a free EC kit. Upon clicking on the study link, participants landed on a study‐specific page on the EC store. Although it was not possible to ensure that participants selected the recommended products, choice was made limited so that only the recommended products offered in this study were visible on the EC store landing page. After 12 weeks, participants were e‐mailed with a link to complete the follow‐up questionnaire, which asked about abstinence and cigarette smoking (cigarettes smoked per day in the last 7 days), EC use, adherence to the advice given and helpfulness of the different intervention components. Those who completed the follow‐up questionnaire were provided with a £10 Amazon voucher. Those who did not complete the follow‐up survey after five e‐mail reminders were e‐mailed and texted one‐single question: ‘Have you smoked at all in the last 4 weeks’ to maximize data for the primary outcome.

### Interventions

The intervention components were:

1. ‘Advice on device’ was tailored using three 5‐point Likert scale items with the options ‘strongly disagree to strongly agree’ scoring from 1 to 5: (i) ‘The e‐cigarette must be small’, (ii) ‘I prefer to be able to see lots of vapour, including when exhaling’ (reverse‐scoring 5 to 1) and (iii) ‘The technicalities of the e‐cigarette put me off’. Participants scoring between 3 and 7 across the three items were recommended to purchase a tank system e‐cigarette device (Arc 5), which is typically associated with greater volume of aerosol. Those scoring between 8 and 11 were assigned a tank system pen‐like device (Tornado EX2), and a refillable pod‐system (Skope‐P) was recommended for those who score 12 or more.
2. ‘Advice on nicotine strength’ was determined from participants’ answers to the question: ‘How soon after waking do you smoke your first cigarette?’, an item making up both the Fagerström Test for Cigarette Dependence (FTCD) [41] and Heaviness of Smoking Index (HSI) [42]. Those who smoked within 30 minutes of waking were recommended starting on 18 mg/ml nicotine concentration, while those who smoked within 30–60 minutes and after 60 minutes of waking were recommended 14 mg/ml and 10 mg/ml, respectively.
3. ‘Advice on flavour’ was tailored using the following: ‘Do you smoke more menthol cigarettes than regular tobacco cigarettes?’. Those answering ‘Yes’ were recommended menthol flavour, and those answering ‘No’ were directed to a question using items that assess taste preferences (i.e. ‘In your attempt to quit smoking, do you want something that tastes like smoking or a complete change?’—with the options: ‘Yes I want something that tastes like smoking’ resulting in being assigned tobacco and ‘No I want a complete change’ resulting in being recommended fruit flavour).
4. Text messages (70 + 2 instructional; co‐created with vapers and smokers) were sent to those allocated to the text message condition using simple mail transfer protocol (SMTP) via PageOne Communications Ltd (no reply), twice daily for the first 2 weeks, one a day for the following 4 weeks, then every other day for 4 weeks, and finally one a week for 2 weeks (the full bank of messages are available via open access) [32]. These covered practical tips around how to maintain your EC device equipment (e.g. remember to charge, change the coil), social support (e.g. talk to experienced vapers, reward yourself) comparing vaping to smoking (e.g. differences in puffing techniques), preventing relapse (e.g. advice to use a higher nicotine strength if using alcohol, do not be afraid of trying different flavours), identity (e.g. emphasizing that vaping can be different for different people) and health and safety (highlighting the relative risk of vaping compared to smoking) [32], as well as some generic smoking cessation texts taken from the iQuit in Practice message bank [32].
5. ‘Brief information on relative harms’ was e‐mailed to participants and provided information about vaping in order to allay safety concerns and misperceptions of harm. We used the Cancer Research UK one‐page infographic on the relative harm of EC use compared to smoking, which was developed in 2018 (see Supporting information, Fig. S1).

### Measures

Participants provided information on demographics, smoking characteristics including smoking dependence [time to first cigarette (TFC) taken from the HSI [43] and years smoked], motivation to quit smoking [using the motivation to stop scale (MTSS) [44]], cessation self‐efficacy, past quit attempts and vaping history.

Perceptions of harms associated with e‐cigarettes was measured using a single‐item 6‐point Likert‐type scale (‘Compared to tobacco smoking, how harmful do you think electronic cigarettes are?’ with the options ‘much more harmful’ to ‘a lot less harmful’) adapted from the Action on Smoking and Health (ASH) survey [45].

To measure adherence to recommendation, participants were asked at follow‐up whether they had purchased (a) the device, (b) the nicotine strength and (c) the flavour we recommended. They were also asked if they had received the e‐mail with the information on relative harms, if they had read it and whether they had blocked the text messages (all answers: yes/no).

#### Outcomes

All participants were prompted to complete a follow‐up questionnaire to assess abstinence at 12 weeks post‐randomization. The primary outcome was the proportion who reported complete abstinence from smoking (not a single puff) over the previous 4 weeks (regardless of EC use). Secondary outcomes were (i) the proportion who reported complete abstinence from smoking over the previous 7 days, (ii) the proportion who reported 50% or greater smoking reduction in baseline cigarette consumption and (iii) adherence to recommendations.

### Analyses

#### Primary analyses

Multivariable logistic regression was conducted using a three‐staged approach to model the primary outcome on an intention to treat basis. First, all main effects, two‐ and three‐way interactions were included. Where there was no evidence of significant three‐way interactions, these were dropped from the model and two‐way interactions were explored. In the case of significant three‐way interactions, intervention components involved in these significant three‐way interactions were excluded from the two‐way interactions analyses. Lastly, we repeated this sequential approach to explore remaining main effects not involved in the significant interactions. All participants who were randomized and met inclusion criteria were included in the analysis (which excluded 241 duplicates and ‘bots’ who were erroneously randomized by the automated system; see further explanation in Supporting information, S3). Participants lost to follow‐up were classified as smoking on the basis of the assumption of missing not at random (MNAR; Little's test, *P* < 0.001), in accordance with the Russell Standard criteria for smoking abstinence [46]. All effects were tested at *P* < 0.05. The model was repeated for the secondary smoking outcomes: 7‐day abstinence at 12 weeks and ≥ 50% smoking reduction.

#### Sensitivity analyses

We repeated the above models excluding those who did not redeem their vouchers (i.e. did not proceed to making their on‐line purchase for an EC kit) for both the primary and secondary smoking outcomes. A further model included only participants who completed the follow‐up survey at 12 weeks (complete case) on the assumption of data being missing completely at random (MCAR; Little's test, *P* = 0.005).

All the above analyses were also repeated to adjust for socio‐demographics and smoking characteristics covariates [these were age, gender, ethnicity, socio‐economic status (SES), MTSS and TFC].

Bayes factor (BF) coefficients were computed for all main effects and interactions (Table 4 for primary analysis) and significant interactions were followed‐up (see Supporting information, S5). For adherence to recommendations, frequencies and percentages are reported.

#### Changes to analysis plan

The above analyses were per protocol [36] except secondary outcome (ii) ≥ 50% smoking reduction. In our protocol we specified that we would test ≥ 50% smoking reduction only in those who did not achieve full abstinence. However, here we present the analysis from the combined group of ≥ 50% reducers, as the original planned analysis may have underestimated success rates by splitting the group into abstainers and reducers. The table of results from the originally specified analyses are available in Supporting information, Table S5. We also indicated in the protocol that we would test time to switch in those who successfully abstained at 12 weeks. However, in the absence of main effects on quitting, this analysis was not conducted.

## RESULTS

### Participants

**FIGURE 1:**
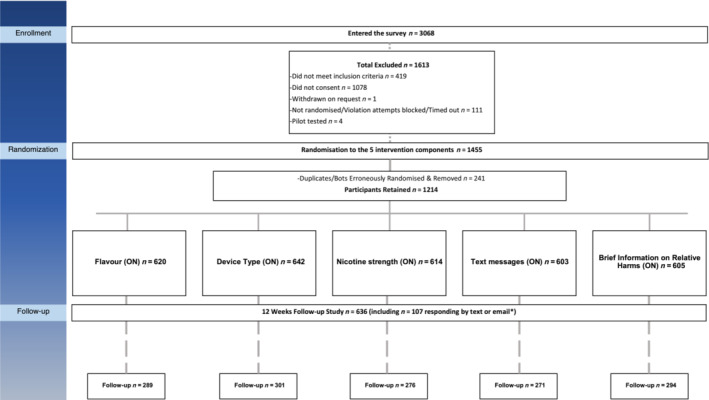
Consolidated Standards of Reporting Trials (CONSORT) diagram for study participation. *Note*: *n* =1214 participants were randomised to one of the 32 computer‐generated block permutations, deriving from the 5 intervention components: 1‐tailored e‐liquid flavour advice, 2‐tailored device selection advice, 3‐tailored eliquid nicotine strength advice, 4‐text message support and 5‐brief information on relative harms, each intervention component ON or OFF (‘ON’ denotes intervention received). Thus, total number of participants in all intervention components combined exceed the overall sample size as participants could be randomised to more than one intervention component at a time (see supplementary file Table S1 for *n* per permutation). *Participants who failed to complete the 12‐week follow‐up survey were asked ‘Have you smoked at all in the last 4 weeks’ via email and text, *n* =107 responses were received.

Figure 1 shows the flow of participants through the study. After removing the 241 bots and duplicates, 1214 eligible participants were randomized. The sample had a mean age of 39 years, 61% were female, 97% white and smoked on average 18 cigarettes per day at baseline. Baseline socio‐demographics and smoking characteristics for the whole sample are presented in Table 1 and by intervention component in Supporting information, Table S2. Overall, there were no differences between conditions except for occupation in nicotine strength, for highest qualification in advice on flavour and device, for past quit attempts in text messages and for smoking history, differences were observed in brief information (see Supporting information, Table S2).

**TABLE 1:** Participants characteristics for the overall sample (*n* = 1214).

	*n* (%)	Mean (SD)	Min–Max
Sex (*n* = 1214)			
Male	467 (39)	–	–
Female	739 (61)	–	–
Other (non‐binary, not disclosed, missing)	8 (1)	–	–
Ethnicity (*n* = 1214)			
White	1171 (97)	–	–
Black/African/Caribbean	3 (0)	–	–
Mixed/multiple ethnic background	19 (2)	–	–
South Asian/Indian/Pakistani/Bangladeshi	10 (1)	–	–
Chinese/Other Asian background	2 (0)	–	–
Other	9 (1)	–	–
Occupation (*n* = 1213)			
Student	97 (8)	–	–
Home carer	115 (10)	–	–
Retired	34 (3)	–	–
Unemployed and looking for work	122 (10)	–	–
Never worked or long‐term unemployed	13 (1)	–	–
Sick and/or disabled	121 (10)	–	–
Routine and manual occupation	313 (26)	–	–
Intermediate occupation	185 (15)	–	–
Managerial and professional occupation	127 (11)	–	–
Self‐employed	86 (7)	–	–
Highest qualification to date (*n* = 1198)			
Degree (or equivalent)	135 (11)	–	–
Higher education (below degree level)	87 (7)	–	–
A‐levels or Highers	209 (17)	–	–
ONC or national level BTEC	99 (8)	–	–
O‐Level or GCSE equivalent (A–C)	302 (25)	–	–
GCSE (D–E), CSE [2–5] or standard grade [4–6]	159 (13)	–	–
Other qualifications	44 (4)	–	–
No formal qualifications	147 (12)	–	–
Past EC^a^ use (*n* = 1213)			
Never used	554 (45)	–	–
Some experimentation^b^	441 (34)	–	–
Former occasional users^c^	157 (13)	–	–
Former daily users	41 (3)	–	–
Current occasional user	50 (4)	–	–
Quit attempts (*n* = 1214)			
Yes (number of past quit attempts: *n* = 589)	608 (50)	2.4 (2.2)	1–30
No	606 (50)	–	–
TFC (HIS)^d^ (*n* = 1214)			
< 5 minutes	593 (49)	–	–
6–30 minutes	483 (40)	–	–
31–60 minutes	95 (8)	–	–
After 60 minutes	43 (4)	–	–
Age (*n* = 1205)	–	38.9 (13.0)	18–75
CPD^e^ (*n* = 1210)	–	17.8 (8.3)	2–125
Number of years smoking (*n* = 1201)	–	19.9 (12.9)	0–58
MTSS^f^ (*n* = 1204)	–	5.3 (1.3)	1–7

^a^ EC = e‐cigarettes.

^b^ Some experimentation includes those who have tried an e‐cigarette (EC) once or twice in the past and no longer use one.

^c^ Former occasional users represents those who used an EC occasionally (not daily) in the past and no longer use one.

^d^ TFC (HSI) = time to first cigarette of the day (from the Heaviness of Smoking Index).

^e^ CPD = cigarettes smoked per day.

^f^ MTSS = motivation to stop (smoking) Scale. ONC = Ordinary National Certificate; BTEC = Business and Technology Education Council; GCSE = General Certificate of Secondary Education.

At follow‐up, 44% (529) of participants completed the survey in full and follow‐up rates did not differ across the five intervention components (see Fig. 1). An additional 107 provided information on the primary outcome via text/e‐mail only. Thus, data on the primary outcome were available for 52% (636 of 1214) of the original sample and imputed as have returned to smoking for the remaining 48% in the primary analyses.

### Adherence and engagement with recommendations

Self‐reported adherence to the recommended EC device, flavour and nicotine strength and engagement with brief information and text messages are presented in Table 2. Adherence/engagement was generally high for all components (> 86%), except for brief information, which only 19% reported reading.

**TABLE 2:** Self‐reported adherence to recommendations for those who were given at least one tailored advice.

Did you follow/engage with our recommendation(s) about the following?	
Intervention components	Yes *n* (%)
Flavour (*n* = ON: 197)	170 (86)
Device (*n* = ON: 209)	192 (92)
Nicotine (*n* = ON: 189)	177 (94)
Text messages (*n* = ON: 216)^a^	202 (94)
Brief information on relative harms (*n* = ON: 232)^b^	45 (19)

^a^ We have calculated the percentage for the text messages component based on the number of those who did not block the text messages, as per their follow‐up responses.

^b^ Denotes the number of people who said they had read the brief information (70% of people reported ‘I did not receive this e‐mail’ and an additional 11% reported they had not read the information).

### Smoking cessation and reduction

**FIGURE 2:**
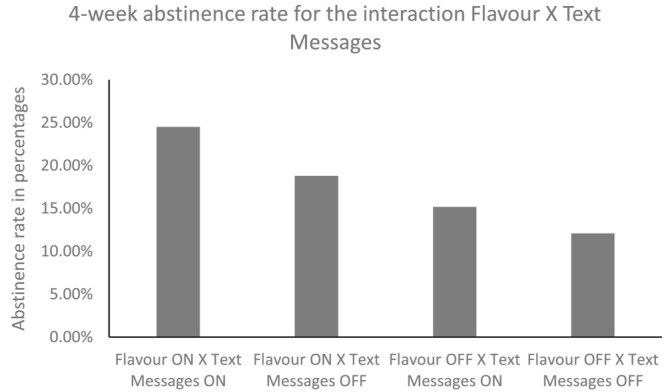
Significant interactions for the primary outcome 4‐week point‐prevalence abstinence at 12 weeks post‐randomization; graph represents the flavour × text messages interaction.

The overall abstinence rate for the primary outcome was 19%. A further 13% reported reducing their cigarette consumption by ≥ 50% with a mean reduction of 83% [standard deviation (SD) = 14.3]. Table 3 presents self‐reported abstinence rates and ≥ 50% smoking reduction across each of the five interventions. Table 4 presents the final model for the primary analysis including the significant interactions and main effects. There were no significant three‐way interactions on the primary or secondary outcomes. However, there was a significant two‐way interaction between flavour and text messages [OR = 1.55, 95% confidence interval (CI) = 1.13–2.14, *P* = 0.007, BF = 7.25]. When advice on flavour and text messages were combined, smokers were more likely to report abstinence over the previous 4 weeks (25%) compared with those who received advice on flavour alone or text message alone (19 and 15%) or those who received neither (12%) (Fig. 2). This interaction was also observed for ≥ 50% smoking reduction and 7‐day point prevalence, but the latter was not significant in the fully adjusted model (*P* = 0.054) (see Table 4). There were no significant main effects.

**TABLE 3:** Self‐reported point prevalence abstinence at 4 weeks, 7 days (*n* = 1214) and ≥ 50% smoking reduction at 12 weeks post‐randomization.

	*n* (%) Abstinence at 4 weeks		*n* (%) Abstinence at 7‐day		*n* (%) ≥ 50% Smoking reduction	
Intervention components	ON^a^	OFF^a^	ON	OFF	ON	OFF
Flavour (*n* = ON: 620; OFF: 594)	136 (22)	101 (17)	142 (23)	119 (20)	210 (34)	184 (31)
Device (*n* = ON: 642; OFF: 572)	116 (18)	114 (20)	135 (21)	132 (23)	205 (32)	183 (32)
Nicotine (*n* = ON: 614; OFF: 600)	120 (20)	114 (19)	129 (22)	126 (21)	190 (31)	204 (34)
Texts (*n* = ON: 603; OFF: 611)	121 (20)	116 (19)	139 (22)	128 (21)	199 (33)	196 (32)
Written information (*n* = ON: 605; OFF: 609)	121 (20)	110 (18)	139 (23)	128 (21)	212 (35)	183 (30)

^a^ ON denotes presence of the intervention component; that is, participants allocated to receive the given intervention component and OFF denotes absence of that given intervention; that is, participants allocated not to receive the intervention component.

**TABLE 4:** Final logistic regression models for the primary analysis: (1) primary outcome 4‐week abstinence at 12 weeks, (2) secondary outcome 7‐day abstinence at 12 weeks and (3) secondary outcome ≥ 50% reduction in smoking from baseline.

	4–weeks abstinence						7–day abstinence						50% smoking reduction					
	Unadjusted			Adjusted			Unadjusted			Adjusted			Unadjusted			Adjusted		
Intervention components	OR (95% CI)	*P*	Bayes factor	OR (95% CI)	*P*	Bayes factor	OR (95% CI)	*P*	Bayes factor	OR (95% CI)	*P*	Bayes factor	OR (95% CI)	*P*	Bayes factor	OR (95% CI)	*P*	Bayes factor
Main effects																		
Flavour	–	–	–	–	–	–	–	–	–	–	–	–	–	–	–	–	–	–
Device	0.89 (0.57–1.19)	0.44	0.58	0.90 (0.67–1.21)	0.48	0.56	0.91 (0.69–1.20)	0.50	0.52	0.93 (0.70–1.23)	0.60	0.49	1.02 (0.80–1.30)	0.90	0.39	1.05 (0.81–1.34)	0.73	0.42
Nicotine	1.08 (0.81–1.44)	0.79	0.50	1.10 (0.82–1.48)	0.52	0.54	1.08 (0.82–1.42)	0.61	0.49	1.10 (0.83–1.46)	0.49	0.53	0.85 (0.66–1.08)	0.18	0.82	0.84 (0.65–1.07)	0.16	0.91
Texts	–	–	–	–	–	–	–	–	–	–	–	–	–	–	–	–	–	–
Brief information on relative harms	1.11 (0.83–1.49)	0.47	0.55	1.13 (0.84–1.51)	0.43	0.59	1.07 (0.81–1.41)	0.63	0.48	1.08 (0.82–1.43)	0.59	0.49	1.21 (0.95–1.55)	0.13	1.03	1.22 (0.95–1.56)	0.12	1.14
2‐way interactions																		
Flavour × texts	**1.56 (1.14–2.14)**	**0.005**	**8.91**	**1.55 (1.13–2.14)**	**0.007**	**7.95**	**1.38 (1.02–1.87)**	**0.040**	**2.52**	1.36 (1.00–1.85)	0.054	2.10	**1.40 (1.06–1.83)**	**0.017**	**5.10**	**1.41 (1.07–1.86)**	**0.016**	**4.78**

The final multivariable logistic regression includes the five main effects and the significant two‐way interaction. A three‐stage approach was used to model the primary (4‐week abstinence at 12 weeks) and the secondary outcomes (7‐day abstinence at 12 weeks and ≥ 50% reduction in smoking). In Stage 1, all main effects, two‐ and three‐way interactions were entered into the models. There were no significant three‐way interactions. Subsequently, in Stage 2, to give final estimates of the significant two‐way interactions, the significant two‐way interactions were entered into the model; finally, in Stage 3, with the model reduced to only significant two‐factor interactions, the main effects of the factors not involved in interactions were entered into the final model. Statistics are reported for the unadjusted and adjusted models [i.e. adjusting for socio‐demographics and smoking characteristics covariates, age, gender, ethnicity, socio‐economic status (SES), MTSS (motivation to stop smoking), and TFC (time to first cigarette)]; statistically significant effects (*P* < 0.05) are shown in bold type. OR (95% CI) = odds ratio (95% confidence intervals).

#### Sensitivity analyses

After removing 12 participants who did not redeem their vouchers, the pattern of results remained similar (see Supporting information, Table S3), with flavour by text the only significant interaction for all, primary and secondary outcomes.

For the complete case analysis, text messages interacted with flavour (OR = 1.62, 95% CI = 1.08–2.42, *P* = 0.019, BF = 3.69) to increase the odds of quitting when both components were delivered together (47%), when advice on flavour was delivered alone (46%) and when both were not provided (42%) compared to text message support delivered alone (31%). The same pattern of findings was observed for ≥ 50% reduction; flavour by text message interacted synergistically to increase odds of reducing consumption, but not for 7‐day point prevalence (see Supporting information, Table S4).

## DISCUSSION

Using a factorial design as per the optimization phase of MOST, none of the five e‐cigarette‐orientated intervention components, when delivered alone, significantly increased the odds of reporting complete abstinence from smoking over the previous 4 weeks at the 12‐week follow‐up. Overall, quit rates were consistently higher when participants received advice on flavour combined with text message support. This combination was consistently observed across all outcomes. There were no other two‐ or three‐way interactions or main effects.

The advantageous effect of flavour advice and text message support combined was found for the primary (4‐week abstinence) and secondary (7‐day abstinence and ≥ 50% reduction) outcomes as well as in the sensitivity analyses, supporting its robustness. Previous studies using on‐line interventions delivered with or without interaction or tailoring [47], text message support [32] or EC provision [48] have reported 4‐week abstinence rates ranging between 9 and 18% at 6 months. The percentage reporting 4‐week abstinence here, when provided with both tailored advice on flavour and text message support (25%), compares favourably with these previous studies even allowing for further relapse between 3 and 6 months [49, 50]. Thus, there is preliminary evidence to suggest that providing tailored advice on choice of flavour together with text message support may improve abstinence rates for smokers who are making an EC purchase on‐line.

While there is existing evidence to demonstrate the efficacy of text message support for smoking cessation [20, 21] this is the first study, to our knowledge, to test text messages designed specifically to support smokers to transition fully to EC use. However, why combining text message support specifically with advice on choice of flavour should promote abstinence is unclear. One possible explanation is that some of the text messages suggested trying different flavours if the flavour was not to the recipient's taste or if they become bored with the flavour. Hence, those who received tailored advice on flavour alone may have thought they should stick with that flavour, and if they did not like it may have discontinued EC use. Although worthy of further investigation, this would suggest that guidance on flavour choice is important initially, but only when accompanied by longer‐term advice encouraging flexible use of flavours.

Offering tailored advice on device type or nicotine strength did not increase quit rates and did not interact significantly with any other intervention components. This could be due to the removal of autonomy over device choice or because the tailoring was oversimplified or not aligned with needs. Our tailoring for device type was based on responses to questions about preference for EC size and vapour production as well as technical capabilities, although we could not control for possible differences in nicotine delivery between the devices or for differences in user behaviour (e.g. some participants may have failed to use their devices optimally); other important factors may have been overlooked. Alternatively, participants may not have provided meaningful answers to our questions to sufficiently inform tailored advice; for example, they may not have known their preferences, in turn finding the questions difficult to answer. It is also possible that incompatible advice was provided regarding the combination of device type and nicotine strength as these were treated independently. That is, our design did not allow us to provide the optimal recommendation of nicotine strength based on device type. Indeed, it is commonly reported that higher‐powered devices are generally used with lower nicotine concentrations compared with lower‐powered devices [51, 52]. Further work in this area might consider providing tailored advice on nicotine strength based on both type of device used and time to first cigarette, and not on these intervention components in isolation.

Brief information on relative harms was not a significant predictor of quit success and did not interact with any of the other intervention components to increase the odds of quitting. The purpose of adding this component was to correct misperceptions around EC use and promote their use to support smoking cessation, as observed previously [53]. However, our null findings do not lend support for this very brief type of intervention as helpful in promoting cessation or smoking reduction. It is worth noting, however, that confidence in these specific findings could be undermined by the reduced sample size, wide CI and poor engagement with brief information (only 19% of those followed‐up reported reading this).

The lack of engagement with the brief information on relative harms could be due to impracticality or disinclination to read an e‐mail attachment. As participants signed up to a study about EC and willingness to use was an inclusion criterion, they may have had low levels of concerns about safety issues and therefore not seen a strong need to read it. Incorrect e‐mail entries (either erroneously or fraudulently) or e‐mails being diverted into the junk/spam folders could also contribute to the low level of engagement. Improving the delivery mechanism and visibility of this information may have yielded different findings, although previous research on print‐based materials for smoking cessation has yielded only modest increases in quit rates [40]. Adherence rates (following our recommendation to choose a particular device, flavour or nicotine strength or not blocking the text messages) to all other intervention components were generally very high, suggesting that participants were engaged with the study, although this information was collected at follow‐up and relied upon retrospective recall. Thus, the extent to which those who did not provide 12‐week follow up data engaged appropriately with the intervention components is unclear.

Despite using methods to prevent fraudulent responding (blocking duplicate IP addresses, captcha, mobile telephone numbers and post‐codes, de‐emphasizing the incentive and clearly stating that multiple responses were not permitted) we observed a number of duplicate entries/bots (17%) which failed to be detected prior to our automated randomization. This is an inherent problem with on‐line research, especially where incentives are offered [54, 55], and the problem has been discussed at length elsewhere [56, 57]. Continuous monitoring throughout the data collection period and thorough screening allowed us to manually remove these fraudulent entries, although it is possible that some duplicates/bots could not be detected, which may undermine the integrity of the data. Nevertheless, it is unlikely that our data were unduly influenced by these entries, as they were equally distributed across the 32 permutations and those who did or did not receive each intervention were broadly similar on key baseline socio‐demographic and smoking‐related characteristics (see Supporting information, Table S2).

Our findings should be taken in the context of the data collection period which was at the beginning of the COVID‐19 pandemic, spanning the first lockdown. At this time, many workers were being furloughed, encouraged to stay at home, vape shops were closed and the ‘quit for COVID’ narrative was prevalent on social media. How this might have influenced motivation and ability to quit is unclear, as competing factors could be at play; for example, the juxtaposition of increased stress and removal of usual cues associated with smoking. Although data were collected on‐line, which led to minimal direct impact on data collection, severe delays in delivery of goods around the country meant that some participants did not receive their EC kits. It is possible that participation could have been driven by dishonest motives, to obtain a free e‐cigarette or simply due to boredom, which may question how meaningful and trustworthy responses were. However, motivation to stop smoking was generally high (mean = 5; ‘I want to stop smoking and hope to soon’) which is consistent with other reports of increased motivation to quit during the COVID pandemic [58]. Nevertheless, there is no reason to expect that the specific pattern of findings observed, involving the controlled experimental manipulation of factors, could be explained by the pandemic. Other limitations include the low follow‐up rate, the lack of biochemical abstinence verification and the short follow‐up period, which should be addressed in future research.

### CONCLUSION

Using a factorial design, guided by the multi‐phase optimization strategy (MOST) to screen multiple intervention components that could be delivered on‐line to support smokers to use e‐cigarettes to quit smoking, our findings suggest that tailored advice on flavour and text message support is a promising intervention combination to promote smoking cessation, and warrants further evaluation in a RCT with biochemically verified abstinence and longer‐term outcomes. These findings highlight the importance of evaluating individual intervention components (main effects and interactions) before embarking on a RCT to maximize the chances of developing an effective and cost‐effective intervention package. Nevertheless, future research is needed to test whether this interaction is replicable, especially as it was not predicted a priori, and data were collected during an atypical period.

## AUTHOR CONTRIBUTIONS

**Catherine Kimber:** Conceptualization (supporting); data curation (lead); formal analysis (equal); funding acquisition (supporting); investigation (equal); methodology (equal); project administration (lead); supervision (equal); validation (equal); visualization (equal); writing—original draft (lead); writing—review and editing (lead). **Vassilis Sideropoulos:** Conceptualization (equal); data curation (supporting); formal analysis (equal); funding acquisition (equal); investigation (supporting); methodology (equal); project administration (supporting); resources (supporting); software (lead); writing—original draft (equal); writing—review and editing (equal). **Sharon Cox:** Conceptualization (equal); formal analysis (supporting); funding acquisition (equal); methodology (equal); writing—review and editing (equal). **Daniel Frings:** Conceptualization (equal); formal analysis (equal); funding acquisition (equal); methodology (equal); writing—review and editing (equal). **Felix Naughton:** Conceptualization (equal); formal analysis (equal); funding acquisition (equal); methodology (equal); writing—review and editing (equal). **Jamie Brown:** Conceptualization (equal); formal analysis (equal); funding acquisition (equal); methodology (equal); writing—review and editing (equal). **Hayden McRobbie:** Conceptualization (equal); formal analysis (equal); funding acquisition (equal); methodology (equal); writing—review and editing (equal). **Lynne Dawkins:** Conceptualization (lead); data curation (equal); formal analysis (lead); funding acquisition (lead); investigation (lead); methodology (lead); project administration (equal); supervision (lead); validation (lead); visualization (lead); writing—original draft (lead); writing—review and editing (lead).

## TRIAL REGISTRATION

Retrospectively registered: ISRCTN54776958; registration date: 21/10/2022.

## DECLARATION OF INTERESTS

C.K., V.S., S.C., D.F., H.M. and F.N. have no conflicts of interest. J.B. has received unrestricted funding to study smoking cessation from pharmaceutical companies who manufacture medically licensed smoking cessations medications (J&J and Pfizer). L.D. has provided consultancy for Johnson & Johnson.

## ETHICS STATEMENT

The study received full ethical approval from London South Bank University's Ethics Panel (approval date: 14 January 2020; reference ETH1920–0043). Participants provided electronic consent prior to completing the baseline survey. All participants were treated in accordance with the Declaration of Helsinki [59].

## Supporting information


**Figure S1.** CRUK Infographics (Supporting Information S2).

**Table S1.** Breakdown of randomisation and permutations for the 5 intervention components: tailored advice on (1) EC device, (2) Nicotine strength, (3) flavour, (4) Brief information, (5) Text messages support.
**Figure S1.** CRUK infographic.
**Figure S2.** CRUK infographic used for the Brief Information condition (See separate file).
**Supporting Information S3.** Explanation of duplicates & bots (at both baseline & 12‐week follow up).
**Table S2.** Participant characteristics broken down by conditions (intervention components on/off for each).
**Supporting Information S5.** Breakdown of interactions for the primary analysis.
**Table S3.** Table of sensitivity analysis 1 (excluding those who did not redeem their vouchers) for primary and secondary outcomes.
**Table S4.** Table of sensitivity analysis 2 (complete case analysis) for primary and secondary outcomes.
**Table S5.** Table for primary analysis on ≥50% reduction as per original plan (excluding quitters).
**Supporting Information S9.** Instructions to participants for the advice tailoring and EC kit purchase.

## Data Availability

The data that support the findings will be available in the LSBU Open research repository at https://openresearch.lsbu.ac.uk/item/92xq8 following an embargo from the date of publication to allow for commercialization of research findings.
