# Supplementary material for: E‐cigarette support for smoking cessation: Identifying the effectiveness of intervention components in an on‐line randomized optimization experiment
Source: Addiction. 2023 Jul 16;118(11):2105–17. doi: 10.1111/add.16294 (PMC10952247; doi:10.1111/add.16294)
Supplement: Supplementary file 1 — Figure S1. CRUK Infographics (Supporting Information S2). [file ADD-118-2105-s002.pdf]

The evidence so far shows that e-cigarettes are far less harmful than smoking

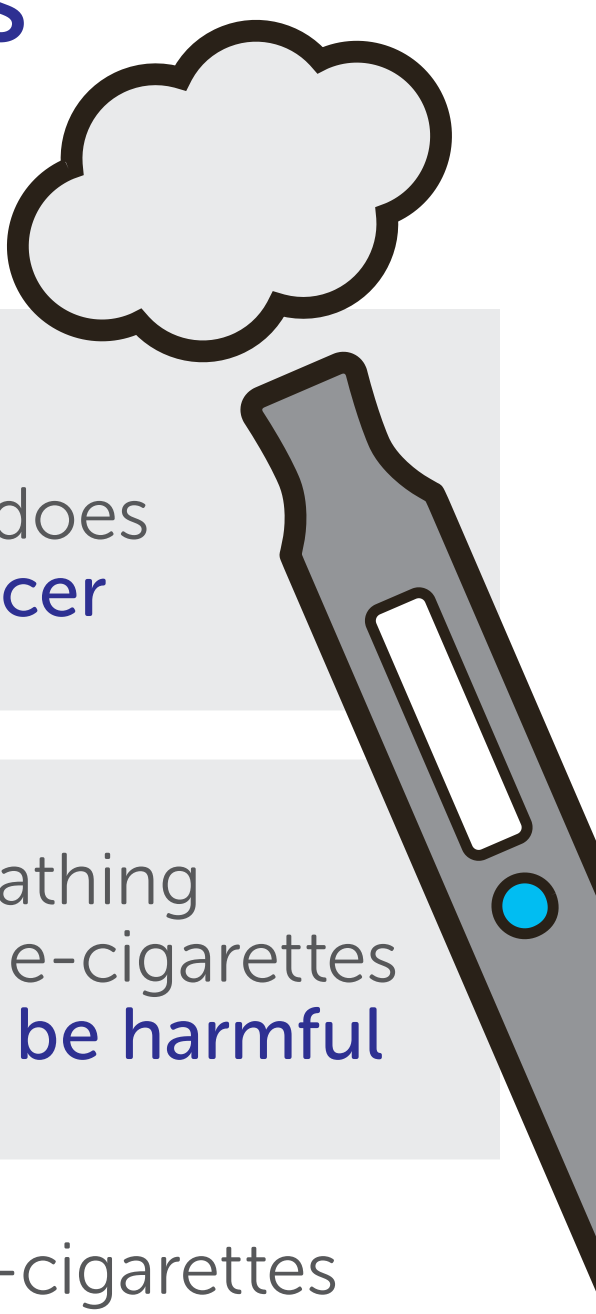

1 E-cigarettes contain nicotine but **not cancer causing tobacco**

2 Nicotine is addictive, but does **not cause cancer**

3 **Tobacco** is the biggest cause of preventable death in the UK

4 Passively breathing vapour from e-cigarettes is **unlikely to be harmful**

5 Growing evidence shows e-cigarettes are helping people to **stop smoking**

? We don't yet know if e-cigarettes have any long-term effects. They are not risk-free and they should not be used by people who haven't smoked.

cruk.org
Together we will beat cancer

CANCER RESEARCH UK
