# Supplementary material for: E‐cigarette support for smoking cessation: Identifying the effectiveness of intervention components in an on‐line randomized optimization experiment
Source: Addiction. 2023 Jul 16;118(11):2105–17. doi: 10.1111/add.16294 (PMC10952247; doi:10.1111/add.16294)
Supplement: Supplementary file 2 — Table S1. Breakdown of randomisation and permutations for the 5 intervention components: tailored advice on (1) EC device, (2) Nicotine strength, (3) flavour, (4) Brief information, (5) Text messages support. Figure S1. CRUK infographic. Figure S2. CRUK infographic used for the Brief Information condition (See separate file). Supporting Information S3. Explanation of duplicates & bots (at both baseline & 12‐week follow up). Table S2. Participant characteristics broken down by conditions (intervention components on/off for each). Supporting Information S5. Breakdown of interactions for the primary analysis. Table S3. Table of sensitivity analysis 1 (excluding those who did not redeem their vouchers) for primary and secondary outcomes. Table S4. Table of sensitivity analysis 2 (complete case analysis) for primary and secondary outcomes. Table S5. Table for primary analysis on ≥50% reduction as per original plan (excluding quitters). Supporting Information S9. Instructions to participants for the advice tailoring and EC kit purchase. [file ADD-118-2105-s001.docx]

**Supporting Information**

| **Table S1. Breakdown of randomisation and permutations for the 5 intervention components: tailored advice on 1) EC device, 2) Nicotine strength, 3) flavour, 4) Brief information, 5) Text messages support** | | | | | | | |
| --- | --- | --- | --- | --- | --- | --- | --- |
| **SPSS coding for permutations** | **EC Device** | **Nicotine strength** | **Flavour** | **Brief info** | **Text messages** | ***n* of Randomised conditions** | ***n* of Randomised conditions (after exclusion of duplicates and bots** |
| 1 | On | On | On | On | On | 46 | 42 |
| 2 | On | On | On | On | Off | 46 | 40 |
| 3 | On | On | On | Off | On | 45 | 32 |
| 4 | On | On | On | Off | Off | 45 | 38 |
| 5 | On | On | Off | On | On | 46 | 41 |
| 6 | On | On | Off | On | Off | 46 | 38 |
| 7 | On | On | Off | Off | On | 46 | 36 |
| 8 | On | On | Off | Off | Off | 46 | 37 |
| 9 | On | Off | On | On | On | 45 | 36 |
| 10 | On | Off | On | On | Off | 45 | 36 |
| 11 | On | Off | On | Off | On | 46 | 42 |
| 12 | On | Off | On | Off | Off | 46 | 39 |
| 13 | On | Off | Off | On | On | 46 | 34 |
| 14 | On | Off | Off | On | Off | 46 | 34 |
| 15 | On | Off | Off | Off | On | 46 | 33 |
| 16 | On | Off | Off | Off | Off | 46 | 44 |
| 17 | Off | On | On | On | On | 46 | 38 |
| 18 | Off | On | On | On | Off | 46 | 40 |
| 19 | Off | On | On | Off | On | 45 | 40 |
| 20 | Off | On | On | Off | Off | 45 | 40 |
| 21 | Off | On | Off | On | On | 46 | 41 |
| 22 | Off | On | Off | On | Off | 45 | 36 |
| 23 | Off | On | Off | Off | On | 46 | 39 |
| 24 | Off | On | Off | Off | Off | 46 | 36 |
| 25 | Off | Off | On | On | On | 46 | 40 |
| 26 | Off | Off | On | On | Off | 46 | 41 |
| 27 | Off | Off | On | Off | On | 46 | 36 |
| 28 | Off | Off | On | Off | Off | 46 | 40 |
| 29 | Off | Off | Off | On | On | 45 | 35 |
| 30 | Off | Off | Off | On | Off | 45 | 33 |
| 31 | Off | Off | Off | Off | On | 46 | 38 |
| 32 | Off | Off | Off | Off | Off | 46 | 39 |
| *Notes. Participants were randomised to one of the 32 conditions (permutations of ON and OFF components of the interventions). For example, for permutation 7: tailored advice for i) device ON, ii) nicotine strength ON, flavour OFF, brief info OFF, text messages ON, participants were advised to select a specific e-cigarette device and nicotine strength (based on their baseline questionnaire responses) and received text messages support. These participants did not receive advice on flavour and brief info. EC denotes E-cigarette; Brief Info denotes brief information on relative harms.* | | | | | | | |

**Figure S2. CRUK infographic used for the Brief Information condition (See separate file)**

**Supp 3. Explanation of duplicates & bots (at both baseline & 12-week follow up)**

*At baseline*

Our inclusion criteria specified that an individual could only take part once. However, despite adding reCAPTCHA and blocking repeat entries from the same IP address, e-mail and phone number, our automated randomisation in Qualtrics failed to detect all duplicates and bots so some individuals were erroneously randomised. Duplicates and bots are protocol violations and were defined as multiple completions from the same individual. These include participants who completed the survey in its entirety multiple times (often in quick succession) thus were randomised to more than one condition. Offending participants managed to circumvent our systems by supplying different email addresses or providing fake telephone numbers. Polite emails were also sent out to repeat offenders to ask them to stop taking the survey and the survey was halted (for a few days) on two occasions to allow us to implement further measures (e.g. blocking repeat post-codes and updating inclusion criteria to only one entry per household). On every instance, only the first entry from that individual was included in the intention-to-treat analysis. All subsequent entries from that same individual (or from the same household unless completions were prior to 1st of April) was removed. Note at the launch of the survey prior to 1st April, completions from different people residing in the same household were permitted. Thus, completions from the same postal address recorded prior to the 1^st^ April where the email address and phone number differed were not counted as violations. Violations were systematically identified as follow:

1. Screening for repeated orders from the retailer where payment was made with the study voucher code (data repository from the retailer)
2. Matching these entries across with Qualtrics datafile
3. In the Qualtrics file looking for duplicate postal, email and IP addresses and/or phone number
4. Reconciling email and IP addresses with the study voucher codes

| **Table S2. Breakdown of participant demographic and smoking-related characteristics per intervention component OFF (component absent) and ON (component received) (N=1214)** | | | | | | | | | | | |
| --- | --- | --- | --- | --- | --- | --- | --- | --- | --- | --- | --- |
| **Intervention Component** | | | | | | | | | | | |
|  | **Flavour** | | | **Device** | | **Nic strength** | | **Brief Info** | | **Text Messages** | |
|  | *Off* | | *On* | *Off* | *On* | *Off* | *On* | *Off* | *On* | *Off* | *On* |
| **Gender (N=1213)** | |  |  |  |  |  |  |  |  |  |  |
| Male, n (%) | | 230 (19) | 237 (20) | 213 (18) | 254 (21) | 238 (20) | 229 (19) | 234 (19) | 233 (19) | 228 (19) | 239 (20) |
| Female, n (%) | | 359 (30) | 380 (31) | 357 (29) | 382 (32) | 357 (29) | 382 (32) | 370 (31) | 369 (30) | 376 (31) | 363 (30) |
| Other, n (%) | | 4 (0) | 3 (0) | 2 (0) | 5 (0) | 4 (0) | 3 (0) | 4 (0) | 3 (0) | 6 (1) | 1 (0) |
| **Ethnicity (n=1214)** | |  |  |  |  |  |  |  |  |  |  |
| White, n (%) | | 567 (47) | 604 (50) | 555 (46) | 616 (51) | 579 (48) | 592 (49) | 588 (48) | 583 (48) | 584 (48) | 587 (48) |
| Other, n (%) | | 27 (2) | 16 (1) | 17 (1) | 26 (2) | 21 (2) | 22 (2) | 21 (2) | 22 (2) | 27 (2) | 16 (1) |
| **Occupation (n=1213)** | |  |  |  |  |  |  |  |  |  |  |
| Student, n (%) | | 47 (4) | 50 (4) | 44 (4) | 53 (4) | 61 (5)* | 36 (3)* | 47 (4) | 50 (4) | 51 (4) | 46 (4) |
| Home carer/Retired, n (%) | | 71 (6) | 78 (6) | 64 (5) | 85 (7) | 73 (6)* | 76 (6)* | 67 (6) | 82 (7) | 73 (6) | 76 (6) |
| Unemployed/Not working^1^, n (%) | | 136 (11) | 120 (10) | 127 (11) | 129 (11) | 111 (9)* | 145 (12)* | 129 (11) | 127 (11) | 120 (10) | 136 (11) |
| Routine & manual, n (%) | | 155 (26) | 158 (26) | 135 (11) | 178 (15) | 145 (12)* | 168 (14)* | 160 (13) | 153 (13) | 155 (26) | 158 (26) |
| Inter/Manag&Prof/Self-employed^2^, n (%) | | 184 (31) | 214 (35) | 201 (17) | 197 (16) | 209 (17)* | 189 (16)* | 206 (17) | 192 (16) | 211 (17) | 187 (15) |
| **Highest qualification (n=1198)** | |  |  |  |  |  |  |  |  |  |  |
| Degree & higher, n (%) | | 88 (7)* | 63 (5)* | 76 (6)* | 75 (6)* | 71 (6) | 80 (7) | 68 (6) | 83 (7) | 81 (7) | 70 (6) |
| A-levels & higher, n (%) | | 188 (16)* | 207 (17)* | 175 (15)* | 220 (18)* | 197 (16) | 198 (17) | 198 (17) | 197 (16) | 189 (16) | 206 (17) |
| GCSE & equivalent, n (%) | | 205 (17)* | 256 (21)* | 208 (17)* | 253 (21)* | 228 (19) | 233 (19) | 239 (20) | 222 (19) | 230 (19) | 231 (19) |
| No formal/other qual, n (%) | | 106 (9)* | 85 (7)* | 106 (9)* | 85 (7)* | 95 (8) | 96 (8) | 95 (8) | 96 (8) | 101 (8) | 90 (8) |
| **Past EC use (n=1213)** | |  |  |  |  |  |  |  |  |  |  |
| Never used, n (%) | | 254 (21) | 300 (25) | 278 (23) | 276 (23) | 278 (23) | 276 (23) | 276 (23) | 278 (23) | 271 (22) | 283 (23) |
| Experimentation^3^, n (%) | | 201 (17) | 210 (17) | 180 (15) | 231 (19) | 197 (16) | 214 (18) | 207 (17) | 204 (17) | 204 (17) | 207 (17) |
| Former occasional users^4^, n (%) | | 86 (7) | 71 (6) | 74 (6) | 83 (7) | 80 (7) | 77 (6) | 80 (7) | 77 (6) | 87 (7) | 70 (6) |
| Former daily users, n (%) | | 22 (2) | 19 (2) | 18 (2) | 23 (2) | 16 (1) | 25 (2) | 18 (2) | 23 (2) | 20 (2) | 21 (2) |
| Occasional users, n (%) | | 30 (3) | 20 (2) | 21 (2) | 29 (2) | 29 (2) | 21 (2) | 27 (2) | 23 (2) | 28 (2) | 22 (2) |
| **Quit attempts (N=1214)** | |  |  |  |  |  |  |  |  |  |  |
| Yes, n (%) | | 296 (24) | 312 (26) | 283 (23) | 325 (27) | 296 (24) | 312 (26) | 310 (26) | 299 (25) | 288 (24)* | 320 (26)* |
| No, n (%) | | 298 (25) | 308 (25) | 289 (24) | 317 (26) | 304 (25) | 302 (24) | 299 (25) | 307 (25) | 323 (27) | 283 (23) |
| ***TFC (HIS)^5^* (N = 1214)** | |  |  |  |  |  |  |  |  |  |  |
| *<5mins,* n (%) | | 285 (24) | 308 (25) | 270 (22) | 323 (27) | 304 (25) | 289 (24) | 292 (24) | 301 (25) | 296 (24) | 297 (25) |
| *6-30 mins,* n (%) | | 243 (20) | 240 (20) | 235 (19) | 248 (20) | 226 (19) | 257 (21) | 254 (21) | 229 (19) | 252 (21) | 231 (19) |
| *31-60 mins*, n (%) | | 41 (3) | 54 (4) | 45 (4) | 50 (4) | 49 (4) | 46 (4) | 43 (4) | 52 (4) | 47 (4) | 48 (4) |
| *After 60 mins*, n (%) | | 25 (2) | 18 (2) | 22 (2) | 21 (2) | 21 (2) | 22 (2) | 20 (2) | 23 (2) | 16 (1) | 27 (2) |
| **Age, Mean (SD) (n=1205)** | | 39.3 (13.1) | 38.5 (13.1) | 39.1 (13.1) | 38.7 (13.1) | 38.8 (13.5) | 38.9 (12.6) | 38.2 (13.1) | 39.6 (13.1) | 39.4 (13.3) | 38.3 (12.9) |
| **CPD^6^, Mean (SD) (n=1210)** | | 17.8 (7.9) | 17.8 (8.7) | 17.6 (7.7) | 18 (8.9) | 17.8 (8.9) | 17.8 (7.7) | 17.6 (7.7) | 18.0 (8.9) | 18.2 (8.9) | 17.4 (7.7) |
| **No of years smoking, Mean (SD) (n=1201)** | | 19.98 (12.9) | 19.7 (12.9) | 19.8 (13.1) | 19.9 (12.8) | 19.6 (13.0) | 20.1 (12.8) | 18.8 (12.8)* | 20.9 (13)* | 20.3 (13.0) | 19.4 (12.8) |
| ***MTSS^7^* Mean (SD) *(n = 1204)*** | | 5.27 (1.34) | 5.31 (1.3) | 5.3 (1.4) | 5.3 (1.3) | 5.3 (1.4) | 5.3 (1.3) | 5.3 (1.3) | 5.3 (1.4) | 5.3 (1.3) | 5.3 (1.3) |
| *Notes. Notes. Chi-Square and T-Tests performed; * denotes significant differences at p≤.05. For ethnicity, occupation and qualification, categories were merged in order to address the issue of small cell count, resulting in fewer categories from that presented in Table 1 for the overall sample characteristics;*  *^1^* ***Unemployed/Not working*** *comprised those reporting being Unemployed and looking for work, those who Never worked or Long-term unemployed, Sick and/or disabled;*  *^2^* ***Interm/Manag& Prof/Self-employed*** *denotes those in the following categories: Intermediate, Managerial & professional occupation and Self-employed*  *^3^* ***Experimentation*** *denotes those who have tried an EC once or twice in the past and no longer use one; ^4^****Former occasional users*** *denotes Used an EC occasionally (not daily) in the past and no longer use one; ^5^****TFC (HIS)*** *denotes Time to First Cigarette of the day (from the Heaviness of Smoking Index); ^6^****CPD*** *denotes Cigarettes per Day; ^7^****MTSS*** *denotes Motivation to Stop [Smoking] Scale* | | | | | | | | | | | |

**Supp 5. Breakdown of Interactions for the Primary Analysis**

**Primary Outcome (4-Week Abstinence)**

Advice on Flavour X Text Messages

1. *Effect of Text if Flavour is ON: χ^2^(1) = 3.00, p= .083; OR = 1.40; p = .084*
2. *Effect of Text if Flavour is OFF: χ^2^(1) = 1.20, p= .273; OR = .786; p = .274*
3. ***Effect of Flavour if Text is ON: χ^2^(1) = 8.36, p= .004; OR = 1.82;*** ***p = .004^a^***
4. *Effect of Flavour if Text is OFF: χ^2^(1) = 0.01, p= .931; OR = 1.02; p = .931*

**Secondary Outcome (7-Day Abstinence)**

Advice on Flavour X Text Messages

1. *Effect of Text if Flavour is ON: χ^2^(1) = 2.00, p= .158; OR = 1.308; p = .159*
2. *Effect of Text if Flavour is OFF: χ^2^(1) = 1.05, p= .307; OR = .811; p = .307*
3. ***Effect of Flavour if Text is ON: χ^2^(1) = 4.67, p= .031; OR = 1.531; p = .032^a^***
4. *Effect of Flavour if Text is OFF: χ^2^(1) = 0.07, p= .794; OR = .950; p = .794*

**Secondary Outcome (≥50% Reduction)**

Advice on Flavour X Text Messages

1. ***Effect of Text if Flavour is ON: χ^2^(1) = 4.41, p= .036; OR = 1.431; p = .036^b^***
2. *Effect of Text if Flavour is OFF: χ^2^(1) = 3.49, p= .0.62; OR = .717; p = .062*
3. ***Effect of Flavour if Text is ON: χ^2^(1) = 7.33, p= .007; OR = 1.606; p = .007^a^***
4. *Effect of Flavour if Text is OFF: χ^2^(1) = 1.57, p= .210; OR = .804; p = .211*

*Footnotes. All significant two-way interactions were broken down; Bold font denotes significant differences at p≤.05; ^a^ For the primary and for both secondary outcomes, the main effect of Flavour was significant when Text was ON, suggesting that smokers who received advice on flavour combined with text message support were more likely to report smoking abstinence in the last 4 weeks, or 7 days or more likely to have reduced their smoking by ≥50%; by contrast those who received advice on flavour or text message alone were less likely to report abstinence. ^b^ For ≥50% smoking reduction outcome, the main effect of Text was significant when Flavour was ON suggesting that text support combined with advice on flavour was more likely to increase the odds of reducing smoking by 50%.*

**Table S3. Final Logistic Regression Models for the first sensitivity analysis (excluding those who did not redeem their vouchers for:** **1) primary outcome (4-week abstinence), 2) secondary outcome 7-day abstinence, and 3) for the secondary outcome ≥50% reduction**

|  | **4-week abstinence** |  |  |  |  |  | **7-Day abstinence** |  |  |  |  |  | **≥50% Smoking Reduction** |  |  |  |  |  |
| --- | --- | --- | --- | --- | --- | --- | --- | --- | --- | --- | --- | --- | --- | --- | --- | --- | --- | --- |
|  | **Unadjusted** |  |  | **Adjusted** |  |  | **Unadjusted** |  |  | **Adjusted** |  |  | **Unadjusted** |  |  | **Adjusted** |  |  |
| **Intervention components** | **OR [95%CI]** | **P** | **Bayes Factor** | **OR [95%CI]** | **P** | **Bayes Factor** | **OR [95%CI]** | **P** | **Bayes Factor** | **OR [95%CI]** | **P** | **Bayes Factor** | **OR [95%CI]** | **P** | **Bayes Factor** | **OR [95%CI]** | **P** | **Bayes Factor** |
| **Main effects** | | | | | | | | | | | | | | | | | | |
| Device | 1.01 [.79-1.29] | .94 | 0.39 | 1.04 [.81-1.24] | .76 | 0.32 | .90 [.69-1.19] | .47 | 0.55 | .92 [.70-1.23] | .58 | 0.51 | 1.01 [.79-1.29] | .94 | 0.39 | 1.04 [.81-1.34] | .76 | 0.42 |
| Nicotine | .85 [.66-1.08] | .19 | 0.82 | .84 [.65-1.08 | .17 | 0.87 | 1.08 [.82-1.42] | .58 | 0.49 | 1.11 [.84-1.47] | .48 | 0.54 | .85 [.66-1.08] | .19 | 0.82 | .84 [.65-1.08] | .17 | 0.87 |
| Brief Info on Relative Harms | 1.21 [.95-1.55] | .12 | 1.03 | 1.22 [.95-1.56] | .12 | 1.14 | 1.07 [.81-1.41] | .63 | 0.48 | 1.08 [.81-1.43] | .60 | 0.49 | 1.21 [.95-1.55] | .12 | 1.03 | 1.22 [.95-1.56] | .17 | 1.14 |
| **2-Way interactions** | | | | | | | | | | | | | | | | | | |
| Flavour X Texts | **1.41 [1.07-1.86]** | **.014** | **4.78** | **1.42 [1.07-1.88]** | **.014** | **4.94** | **1.39 [1.02-1.89]** | **.035** | **2.62** | **1.37 [1.00-1.87** | **.049** | **2.18** | **1.41 [1.07-1.86]** | **.014** | **4.78** | **1.42 [1.07-1.88]** | **.014** | **4.94** |
| *Notes.*  *Multivariable logistic regression including the main effects and the two-way interactions was used to model the primary and secondary outcomes excluding those who did not redeem their vouchers (i.e., did not proceed to making their online purchase for an EC kit). The final model includes the significant main effects and significant two-way interaction following the 3-staged approach. In Stage 1, all main effects, 2- and 3-way interactions were entered into the models. There were no significant 3-way interactions. Subsequently, in Stage 2, to give final estimates of the significant 2-way interactions, the significant 2-way interactions were entered into the model, finally in Stage 3, with the model reduced to only significant 2-factor interactions, the main effects of the factors not involved in interactions were entered into the final model.*  *Statistics are reported for the unadjusted and adjusted models (i.e. to adjust for socio-demographics and smoking characteristics covariates, age, gender, ethnicity, socio-economic status [SES], MTSS [Motivation to Stop Smoking], and TFC [Time to First Cigarette]); Statistically significant effects (p<0.05) are in bold font. OR [95%CI] = Odds ratio [95% Confidence Intervals].* *Dashes indicate intervention components wherein these were not included in the model if involved in the significant interactions* | | | | | | | | | | | | | | | | | | |

**Table S4. Final Logistic Regression Models for sensitivity analysis 2 (complete case analysis – excluding those without 12-week follow up data) for: 1) for the primary outcome (4-week abstinence), 2) secondary outcome 7-day abstinence, and 3) secondary outcome ≥50% reduction**

|  | **4-weeks abstinence** |  |  |  |  |  | **7-Day abstinence** |  |  |  |  |  | **≥50% Smoking Reduction** |  |  |  |  |  |
| --- | --- | --- | --- | --- | --- | --- | --- | --- | --- | --- | --- | --- | --- | --- | --- | --- | --- | --- |
|  | **Unadjusted** |  |  | **Adjusted** |  |  | **Unadjusted** |  |  | **Adjusted** |  |  | **Unadjusted** |  |  | **Adjusted** |  |  |
| Intervention components | OR [95%CI] | P | Bayes Factor | OR [95%CI] | P | Bayes Factor | OR [95%CI] | P | Bayes Factor | OR [95%CI] | P | Bayes Factor | OR [95%CI] | P | Bayes Factor | OR [95%CI] | P | Bayes Factor |
| **Main effects** | | | | | | | | | | | | | | | | | | |
| Flavour | - | - | - | - | - | - | 1.30 [.93-1.83] | .12 | 1.18 | 1.23 [.86-1.76] | .27 | 0.84 | - | - | - | **-** | **-** | **-** |
| Device | **-** | **-** | **-** | **-** | **-** | - | .80 [.57-1.13] | .21 | 0.93 | .79 [.55-1.13] | .20 | 0.96 | .98 [.68-1.41] | .89 | 0.54 | 1.03 [.70-1.52] | .88 | 0.57 |
| Nicotine | - | - | - | - | - | - | 1.28 [.91-1.79] | .16 | 1.10 | 1.36 [.95-1.95] | .10 | 1.45 | .84 [.52-1.21] | .35 | 0.73 | .80 [.54-1.17] | .25 | 0.87 |
| Texts | - | - | - | - | - | - | 1.15 [.82-1.61] | .41 | 0.65 | 1.14 [.80-1.63] | .46 | 0.64 | - | - | - | - | - | - |
| Brief Info on Relative Harms | 1.00 [.71-1.40] | 0.98 | 0.51 | 1.05 [.73-1.50] | .80 | 0.54 | .95 [.68-1.33] | .78 | 0.52 | .95 [.67-1.36] | .79 | 0.55 | 1.21 [.84-1.74] | .31 | 0.78 | 1.31 [.89-1.92] | .17 | 1.08 |
| **2-Way interactions** | | | | | | | | | | | | | | | | | | |
| Flavour X Texts | **1.73 [1.18-2.53]** | **.005** | 8.86 | **1.62 [1.08-2.42]** | **.019** | 3.69 | - | - | - | - | - | - | **1.85 [1.18-2.87]** | **.007** | **6.55** | **1.83[1.14-2.91]** | **.012** | **4.52** |
| Device X Nicotine | 1.10 [.76-1.60] | .62 | 0.60 | 1.24 [.60-2.57] | .56 | 0.84 | - | - | - | - | - | - | - | - | - | - | - | - |
| *Notes. Multivariable logistic regression was used to model the primary and secondary outcomes excluding those who did not complete the 12-week follow-up survey; The final model includes the five main effects and the significant two-way interaction following a 3-staged approach. In Stage 1, all main effects, 2- and 3-way interactions were entered into the models. There were no significant 3-way interactions. Subsequently, in Stage 2, to give final estimates of the significant 2-way interactions, the significant 2-way interactions were entered into the model, finally in Stage 3, with the model reduced to only significant 2-factor interactions, the main effects of the factors not involved in interactions were entered into the final model.*  *Statistics are reported for the unadjusted and adjusted models (i.e. to adjust for socio-demographics and smoking characteristics covariates, age, gender, ethnicity, socio-economic status [SES], MTSS [Motivation to Stop Smoking], and TFC [Time to First Cigarette]); Statistically significant effects (p<0.05) are in bold font. OR [95%CI] = Odds ratio [95% Confidence Intervals].* *Dashes indicate intervention components wherein these were not included in the model if involved in the significant interactions* | | | | | | | | | | | | | | | | | | |

**Table S5. Primary analysis on the secondary outcome ≥50% smoking reduction excluding complete quitters (as per protocol)**

| **50% Smoking Reduction** | | | | | | |
| --- | --- | --- | --- | --- | --- | --- |
|  | **Unadjusted** |  |  | **Adjusted** |  |  |
| **Intervention components** | **OR [95%CI]** | **p value** | **Bayes Factor** | **OR [95%CI]** | **p value** | **Bayes Factor** |
| **Main effects** | | | | | | |
| Texts | 1.19 [.76-1.85] | .45 | 0.74 | 1.18 [.74-1.88] | .49 | 0.73 |
| **3-Way interactions** | | | | | | |
| Flavour X Device X Nicotine | .60 [.23-1.57] | .30 | 0.99 | .58 [.20-1.71] | .32 | 0.98 |
| Flavour X Device X Brief Info | .76 [.29-1.94] | .56 | 0.89 | .86 [.31-2.33] | .76 | 0.88 |
| Flavour X Nicotine X Brief Info | 2.91 [.92-9.15] | .07 | 1.25 | 2.65 [.80-8.76] | .11 | 1.14 |
| *Notes. Multivariable logistic regression including three 3-way interactions (significant in stage 1 of the exploratory approach) and the remaining one main effect to model the secondary outcome ≥50% smoking reduction excluding those who reported complete abstinence. There were no 2-way interactions included in the model as during the exploratory stage there were all involved in the significant 3-way interactions.*  *Statistics are reported for the unadjusted and adjusted models (i.e. to adjust for socio-demographics and smoking characteristics covariates, age, gender, ethnicity, socio-economic status [SES], MTSS [Motivation to Stop Smoking], and TFC [Time to First Cigarette]); Statistically significant effects (p<0.05) are in bold font. OR [95%CI] = Odds ratio [95% Confidence Intervals].* | | | | | | |

**Supp 9. Instructions to participants to facilitate conditions allocation (incl. the advice tailoring) and to guide EC kit purchase process**

The five conditions (ON or OFF) were randomised in Qualtrics using the Randomizer function. As part of the advice tailoring, recommendations regarding the interventions allocated were displayed on the screen at the end of the baseline survey and were also sent via email along with detailed instructions and a direct link to an online EC store with a unique voucher code, purchased by the study team, to receive a free EC kit.

Found below **the instructions (text and graphical images) displayed to participants for each tailored advice in bold** and in *italics the scoring and steps used to tailor the advice*.

## *1 - For those allocated to receive advice on Device type. ‘Advice on device’ was tailored using three five-point Likert scale items with the options “strongly disagree to strongly agree” scoring from 1 to 5: i) “The e-cigarette must be small”, ii) “I prefer to be able to see lots of vapour, including when exhaling” (reverse-scoring 5 to 1) and iii) “The technicalities of the e-cigarette put me off”. Participants* *scoring between 3 and 7 across the three items were recommended to purchase a tank system e-cigarette device (Arc 5) which is typically associated with greater volume of aerosol. Those scoring between 8 and 11 were assigned a tank system pen-like device (Tornado EX2), and a refillable pod-system (Skope-P) was recommended for those who score 12 or more:*

1. Those scoring 12 or more were shown the following text and image:

**Please add the Skope P e-cigarette to your basket - Remember to add spare pods to your basket**


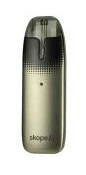


b) Those scoring *between 8 and 11 were assigned a tank system pen-like device (Tornado EX2) and shown the following text and image*:

**Please add the Tornado EX2 to your basket**


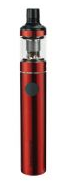


1. Those *scoring between 3 and 7 were recommended to purchase a tank system e-cigarette device (Arc 5)* and were shown the following text and image:

**Please add the arc 5 to your basket**


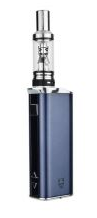


## *2 – For those allocated to receive advice on Nicotine strength. ‘Advice on nicotine strength’ was determined from participants’ answers to the question ‘How soon after waking do you smoke your first cigarette?’ an item making up both the Fagerström Test for Cigarette Dependence [FTCD] and Heaviness of Smoking Index [HIS]. Those who smoked within 30 minutes of waking were recommended to purchase 18mg/ml nicotine concentration, whilst those who smoked within 30-60 minutes and after 60 mins of waking were recommended 14mg/ml and 10mg/ml, respectively.*

1. Participants with a score of 2 or 3 (How soon after you wake up do you smoke your first cigarette?) were shown the following text:

**Please choose the following e-liquid nicotine strength from the ‘Red label’ range 1.8% (18mg/ml) Red label (Note you may see conflicting advice about which nicotine strength to purchase on the website but please ensure you follow our advice)**

1. Participant with a score of 1 were shown the following text:

**Please choose the following e-liquid nicotine strength from the ‘Red label’ range 1.4% (14mg/ml) Red label (Note you may see conflicting advice about which nicotine strength to purchase on the website but please ensure you follow our advice)**

1. Participant with a score 0 on HIS were shown the following text:

**Please choose the following e-liquid nicotine strength from the ‘Red label’ range 1.0% (10mg/ml) Red label (Note you may see conflicting advice about which nicotine strength to purchase on the website but please ensure you follow our advice)**

## *3 – For those advice on Flavour. ‘Advice on flavour’ was tailored using the following: “Do you smoke more menthol cigarettes than regular tobacco cigarettes?”. Those answering ‘Yes’ were recommended menthol flavour, and those answering ‘No’ were directed to a question using items that assess taste preferences (i.e. “In your attempt to quit smoking, do you want something that tastes like smoking or a complete change?” – with the options “Yes I want something that tastes like smoking” resulting in being assigned tobacco and “No I want a complete change” resulting in being recommended fruit flavour).*

1. Participants who answered yes to the question *“Do you smoke more menthol cigarettes than regular tobacco cigarettes?” were shown* the following text:

**Please choose menthol flavour**

b) *Participants who selected the response option “Yes I want something that tastes like smoking” to the question “In your attempt to quit smoking, do you want something that tastes like smoking or a complete change?” were shown the* following text:

**Please choose tobacco flavour**

c) *Those who selected the response option “No I want a complete change” to the question “In your attempt to quit smoking, do you want something that tastes like smoking or a complete change?”* were shown the following text:

**Please choose fruit flavour**

## *4. For those allocated to receive Brief Info, the below text was shown. Participants were emailed the CRUK infographics (see Figure S1).*

**Please take the time to look at the information sheet on smoking and vaping that we will email to you.**

## *5. Those allocated to receive Text messages support were shown the following text (see link to the* [*paper containing the text messages*](https://www.qeios.com/read/W0GEL2)*).*

**Thank you for providing us with your mobile telephone number. We will use this number to send you texts for 3 months about smoking and vaping (using an e-cigarette) to support you in your quit attempt.**

*6. Following completion of the survey, all participants received instructions via email to guide their EC purchase (see below).*

**Thank you for completing our questionnaire.**

**Important: please take a minute to read this information.  We strongly recommend that you open the link to the e-cigarette store in a new browser and keep this email open so you can view your voucher code and assigned product(s). If you have any questions or problems with your voucher code please email us on** [**sasecig@lsbu.ac.uk**](mailto:sasecig@lsbu.ac.uk)

**Your voucher code and the link to the online e-cigarette store can be found at the bottom of this email.  The voucher will entitle you to purchase an e-cigarette starter kit and liquid up to the value of £50.  Please note, use your voucher code within 1 week as this voucher will expire, and the voucher must be used all in one go (i.e. you cannot save any left-over credit for a later purchase).  If you have any credit left over, you can use this in the same transaction to purchase additional e-liquid and/or atomisers.**

**When you click on the link below, this will take you to the online e-cigarette store.  Please start by selecting a vape kit (e-cigarette device) and the colour you want and add it to your basket.  Note, your e-cigarette device will come with one free bottle of e-liquid; you can choose the strength and flavour at checkout.  If you have been assigned a flavour and/or strength, please make sure you follow this advice when you are prompted to select these. Before check-out, you can click on ‘continue shopping’ to select additional e-liquid, atomisers or pods (if you have been assigned the *Skope P*).  We suggest that you add a spare atomiser (or pod, if you have been assigned the *Skope P* device) and at least 1 more bottle of the same e-liquid to your basket - this should last for one to two weeks, after which you will need to purchase more e-liquid.  If you are assigned one or various products (based on your answers to our questions), please ensure you add this assigned product (s) to your basket.  If you are assigned a specific nicotine strength, please choose your e-liquid from the *Red Label* range. If you are not assigned a product, you are free to choose from the product range yourself.**

**Please enter your voucher code at check out.  At this point you will be asked to sign up as a customer with the e-cigarette provider so that your purchase can be shipped out to you. You will need to enter your full details and date of birth in order to verify your age for the purchase. Note: once you register you will see a welcome page with a different offer, please ignore this as your offer as part of this study is significantly better and these offers cannot be combined.**

**When your e-cigarette arrives, please follow the inbox instructions and use your e-cigarette straight away in an attempt to quit smoking.**

**Details of your assigned products are below** *(for those allocated to a condition(s) only)***:**

*RECOMMENDATIONS HERE*

**Go to the site via the link** [**https://www.totallywicked-eliquid.co.uk/lsbu-starter-kits?dsad**](https://www.totallywicked-eliquid.co.uk/lsbu-starter-kits?dsad)

**Voucher Code:  XXXX**
